# Application of Novel Machine Learning Techniques for Predicting the Surface Chloride Concentration in Concrete Containing Waste Material

**DOI:** 10.3390/ma14092297

**Published:** 2021-04-29

**Authors:** Ayaz Ahmad, Furqan Farooq, Krzysztof Adam Ostrowski, Klaudia Śliwa-Wieczorek, Slawomir Czarnecki

**Affiliations:** 1 Department of Civil Engineering, COMSATS University Islamabad, Abbottabad Campus 22060, Pakistan; ayazahmad@cuiatd.edu.pk; 2 Faculty of Civil Engineering, Cracow University of Technology, 24 Warszawska Str., 31-155 Cracow, Poland; krzysztof.ostrowski.1@pk.edu.pl (K.A.O.); klaudia.sliwa-wieczorek@pk.edu.pl (K.Ś.-W.); 3 Faculty of Civil Engineering, Wroclaw University of Science and Technology, Wybrzeze Wyspianskiego 27, 50-370 Wroclaw, Poland

**Keywords:** surface chloride concentration, individual algorithm, aggressive ions environment, gene expression programming, concrete, artificial neural networks

## Abstract

Structures located on the coast are subjected to the long-term influence of chloride ions, which cause the corrosion of steel reinforcements in concrete elements. This corrosion severely affects the performance of the elements and may shorten the lifespan of an entire structure. Even though experimental activities in laboratories might be a solution, they may also be problematic due to time and costs. Thus, the application of individual machine learning (ML) techniques has been investigated to predict surface chloride concentrations (C_c_) in marine structures. For this purpose, the values of C_c_ in tidal, splash, and submerged zones were collected from an extensive literature survey and incorporated into the article. Gene expression programming (GEP), the decision tree (DT), and an artificial neural network (ANN) were used to predict the surface chloride concentrations, and the most accurate algorithm was then selected. The GEP model was the most accurate when compared to ANN and DT, which was confirmed by the high accuracy level of the K-fold cross-validation and linear correlation coefficient (R2), mean absolute error (MAE), mean square error (MSE), and root mean square error (RMSE) parameters. As is shown in the article, the proposed method is an effective and accurate way to predict the surface chloride concentration without the inconveniences of laboratory tests.

## 1. Introduction

**Figure 1:**
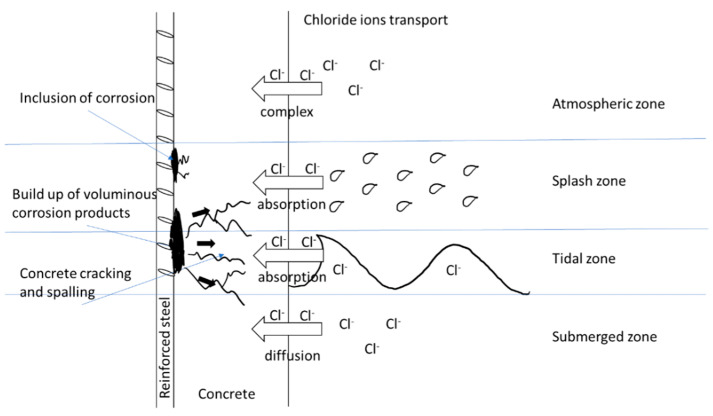
Corrosion process caused by an aggressive chloride environment.

Reinforced concrete (RC) structures are known for their longevity and resistance, which are very valuable in civil engineering practices [1]. This includes the construction of harbor docks, marine structures, and coastal roads. Concrete structures with both steel and composite reinforcement provide strong stability against corrosive action in an alkaline environment [2]. This is mainly due to the massive passive oxide film around reinforcing steel, which can be affected by the presence of chloride ions in sea and coastal structures [3]. These ions first accumulate on the surface of a RC structure and then slowly penetrate into the concrete element [4]. This ultimately demolishes the oxide film and provokes the corrosion of steel, which, in turn, leads to spalling and cracking of the concrete and the reduction of the load-carrying capacity of RC structures [5]. This process significantly reduces the serviceability of an RC structure, causing it to not last for the amount of time for which it was designed. The corrosion process of steel bars embedded in porous concrete is presented in Figure 1. Surface chloride concentrations affect the corrosion of steel and the performance of entire buildings and have a devastating effect in civil engineering [6,7,8]. The durability of a structure is an important parameter in RC buildings for maintaining an adequate service life of concrete structures [9]. Thus, predicting the design service life of concrete structures has become more popular in recent times. However, all prediction models are different and depend on influential factors and the destruction mechanisms of structures [10].

The appearance of chloride ions is a major issue in marine environments and has a very negative effect on RCC structures [11,12]. The possible transmission mechanism of chloride ions into concrete is dependent on the zone where the element is, i.e., the tidal, splash, submerged, and atmospheric zones [13]. The transport of chloride ions in concrete is mainly due to diffusion or the absorption mechanism [14]. The diffusion mechanism that occurs in the submerged zone is due to the saturation of the concrete. However, the absorption mechanism takes place in the tidal and splash zones. This allows the transport of chloride ions into RCC structures. However, the ingress of chloride ions in the atmospheric zone is quite complex when compared to the other zones [15]. This is because of factors associated with the zone, such as the direction and speed of the wind, the salinity level of the water reservoir, and the distance between the sea and the RCC structures. This study focuses on three zones, and omits the atmospheric zone due to its previously described limitations.

The ingress movement of chloride ions in RCC structures is calculated using Fick’s second law of diffusion, as shown in Equation (1). This is mostly used when designing the service life of structures located in a marine environment [16].

(1) $$C\left(x,t\right)=C_{o}+\left(C_{s}-C_{o}\right)\left[1-\mathrm{Erf}\left(\frac{x}{2\sqrt{D\cdot t}}\right)\right]$$

where

*C(x,t)*—chloride concentration at distance (*x*) from the surface after the time of exposure (*t*), mol/m^3^;

*C_o_*—concentration of chloride ions in concrete at the initial stage of their occurrence, mol/m^3^;

*x*—depth from the exposed concrete surface, mm;

*D*—coefficient of apparent chloride diffusion, mm^2^/s;

*C_s_*—apparent surface chloride amount, mol/m^3^;

Erf—error function;

*C_o_* is constant and is not affected by any type of concrete. However, the movement of chloride ions in the marine environment is determined by *C_s_* and *D*. The coefficient of apparent chloride diffusion is a material property that depends primarily on time. It can be determined based on information referring to the composition and microstructure of a material. *C_s_* concentration in diffusion law has a complex nature, as it not only depends on a material’s properties, but also on the environmental conditions and time. This creates ambiguity when making an accurate prediction of chloride ingress in a marine environment. Therefore, studies are needed to build a strong model that uses machine learning approaches, which can accurately predict the amount of apparent surface chloride.

The aim of this study is to predict the surface chloride concentration in marine structures through the application of the gene expression programing algorithm. The levels of accuracy of GEP, DT and an ANN were evaluated and compared in order to choose the most accurate algorithm for the purpose of the study. The most effective algorithm was GEP when compared to DT and ANN. Statistical analyses and K-fold cross validation were used to check the accuracy and validity of all the models. The usability of the proposed algorithm was also compared with other algorithms in the literature, which was important for the purpose of this research.

## 2. Materials and Methods

### 2.1. Apparent Surface Chloride Content

**Figure 2:**
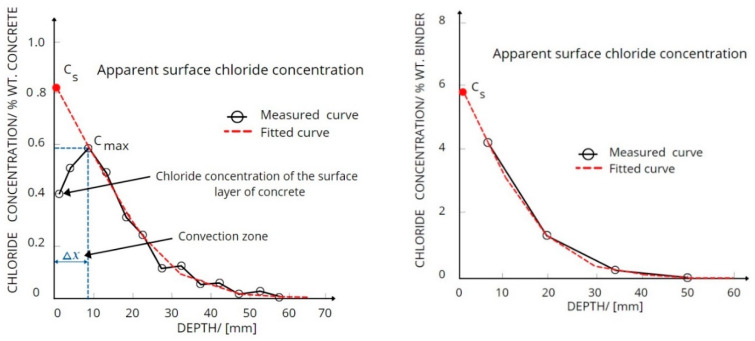
Schematic diagrams for determining *C_s_* under different conditions.

The value of surface chloride concentration *C_s_* is an important variable when describing the transmission of chloride into structures [17]. It is obtained on site, or during laboratory investigations, as depicted in Figure 2. It can be seen that a convention zone is present in the investigated concrete element of the marine structure; however, there is no such zone in the concrete element investigated during the laboratory tests. The value of *C_s_* was obtained through the use of the bulk diffusion profile of chloride using a fitting curve. This variable is the most significant, as it describes the aggression of chloride, quantitative durability, and the prediction of service life of RCC structures.

It is worth mentioning that the *C_s_* value used in the calculations is considered to be constant for the zone in which an element is located. This creates uncertainty due to the complex nature of chloride ion transmission, as it depends on many factors, such as material properties (cement composition, binder properties, and water-to-cement ratio), and environmental factors (zonation, chloride content, depth, relative humidity, and temperature). Many attempts have been made to predict apparent chloride concentrations using logarithmic and exponential functions, or by correlating a concentration with the binder-to-water ratio, material variables, and environmental effects. However, there is still no accurate prediction model that is based on only a small number of variables. In contrast, when using machine learning algorithms, prediction models are more accurate and might be successfully used [18]. In this article, 642 data samples obtained from the literature survey [19] were used to predict surface chloride concentrations through the use of machine learning algorithms.

### 2.2. Machine Learning (ML) and Ensemble Learning (EL) Approaches

Machine learning (ML) is used as an efficient way to predict the mechanical properties of concrete, and its exemplary use is illustrated in Table 1. ML algorithms are more effective than simple correlation models due to the fact that they use the values of more than one variable to predict surface chloride concentrations. Artificial neural networks (ANN), the decision tree (DT), support vector machines (SVM), random forests (RF), gene expression programming (GEP), and deep learning (DL) are the most common algorithms for analyzing the mechanical properties of concrete [20]. Behnood et al. [21] used an ANN with an optimizer as a multi-objective grey wolves (MOGW) model for predicting the mechanical response of silica fume concrete. Getahun et al. [22] used an ANN algorithm to very accurately predict the compressive strength and tensile strength of waste concrete. Ling et al. [23] predicted the compressive strength of concrete in marine environments using SVM and then compared the obtained results with ANN and DT models. It was proved that the SVM was the most accurate. Zaher et al. [24] predicted the compressive property of lightweight foamed concrete using various machine learning techniques. The authors concluded that the extreme learning machine (ELM) was the most accurate and it was successfully applied for predicting the compressive strength of concrete. Woubish et al. [25] used a machine learning approach for the assessment of the durability of reinforced concrete structures. The author revealed that machine learning techniques are useful and that they play a substantial role in predicting the durability of structures when compared to functional CO_2_ and Cl^‒^ ingress models. Suguru et al. [26] developed a model to automatically detect cracks in concrete structures with the use of machine learning. Photographs of concrete structures were used as learning data, and then deep learning was used to detect the cracks. Similarly, Wassim et al. [27] indicated that machine learning models have a high level of accuracy.

### 2.3. Description of the Obtained Data

**Figure 3:**
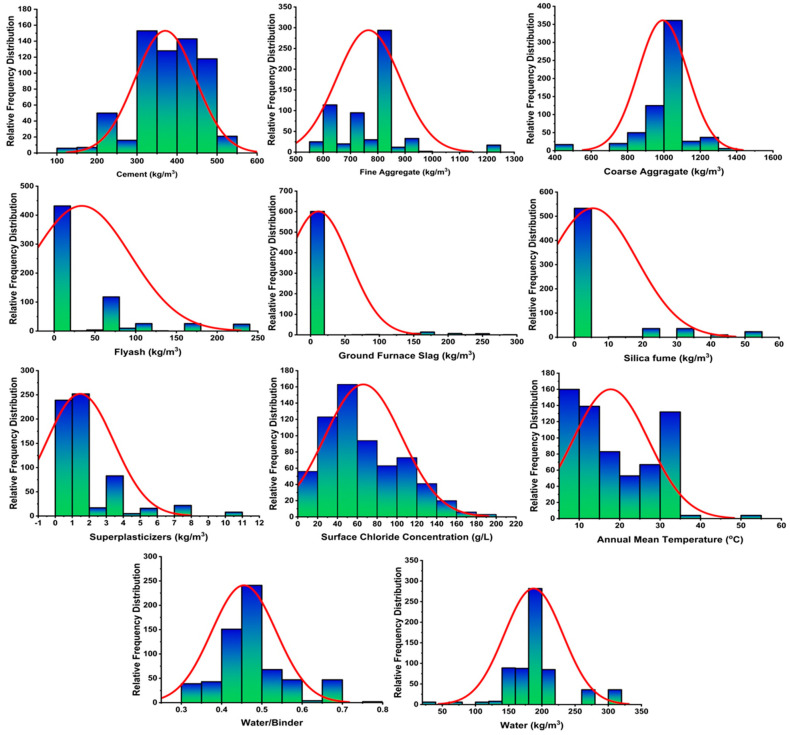
The distribution of input parameters used in the prediction of models.

The data used to model the prediction of the surface chloride concentration are taken from published literature. These data were taken from articles describing the surface chloride concentrations in the tidal zone [48,49,50,51,52,53,54,55,56,57,58,59,60,61,62], splash zone [49,50,54,55,60,63,64] and submerged zone [54,56,60,65,66,67,68]. The data consist of 12 inputs (cement, fine and coarse aggregate, silica fume, fly ash, blast furnace slag, superplasticizer, water, exposure time, annual mean temperature, chloride content, and exposure time) and one output (surface chloride concentration). Jupitar python was used to describe the distribution of each input parameter that was applied in the prediction model and is presented in Figure 3. It is well stated that the performance of a model is significantly affected by its variables [69]. The data variables that were used for modeling, with their ranges, are listed in Table 2 and Table 3.

### 2.4. Machine Learning Algorithms

**Figure 4:**
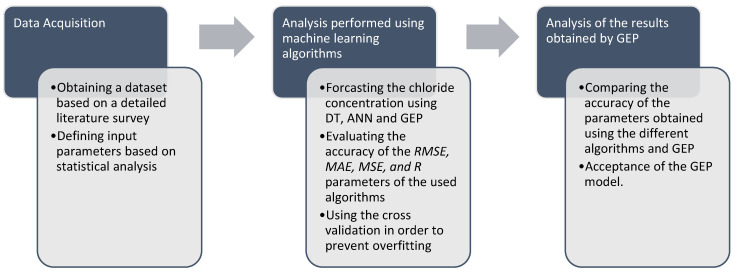
Flowchart of the research approach.

This section describes the algorithms used when modeling the prediction of surface chloride concentrations in the concrete elements of marine structures. The prediction of *C_s_* was made using ANN, DT and GEP. A detailed flowchart of the used methodology is presented in Figure 4.

The decision tree algorithm is based on a classification technique with supervised learning and is used to solve various computational problems with both a regression and classification nature. The tree-like structure of the decision tree can be used to solve the problem. In this algorithm, the nodes are divided into two consecutive sub-nodes up to the end nodes, which, in turn, determine the shape of the decision tree. The identification of the attributes from the root node at each level is considered to be a challenging task when using the decision tree algorithm. The whole procedure is called “the selection of attributes”. The division in the nodes is made on the basis of criteria. In the case of regression issues, the division is made by determining the point of separation, while in the case of classification issues, the criterion for division is the value of one of the classes. Different learning algorithms were used to split the nodes in order to obtain a convergence of the results. Moreover, a convergence of the results can also be obtained by using *n* number of trees in the algorithm.

**Figure 5:**
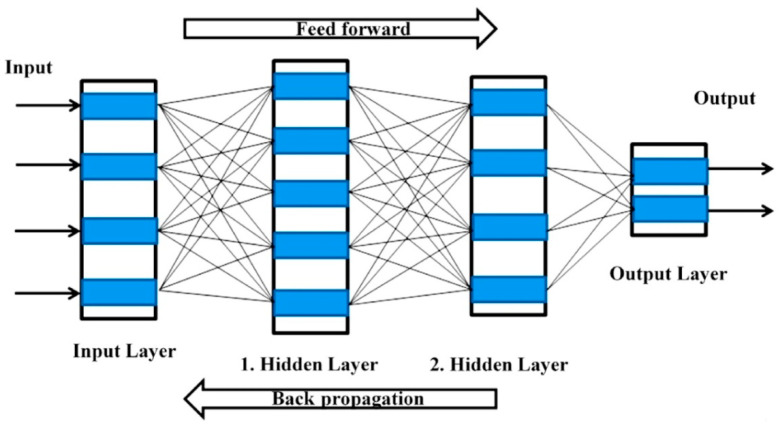
Scheme of the ANN.

ANN is considered to be one of the most popular machine learning algorithms. An ANN can be used for learning, predicting and making decisions based on input data. The basic datasets for ANN models include training, testing, and validation. During the training process, the ANN learns based on the patterns of the prediction model. The validation process evaluates the accuracy of the trained model. According to the literature survey, the feed forward and the feed forward back propagation (FFBP) neural networks are the most commonly used when solving engineering problems [70]. These types of ANN consist of input, hidden and output layers that contain neurons [71]. It is activated using the activation function, as can be seen in Figure 5.

**Figure 6:**
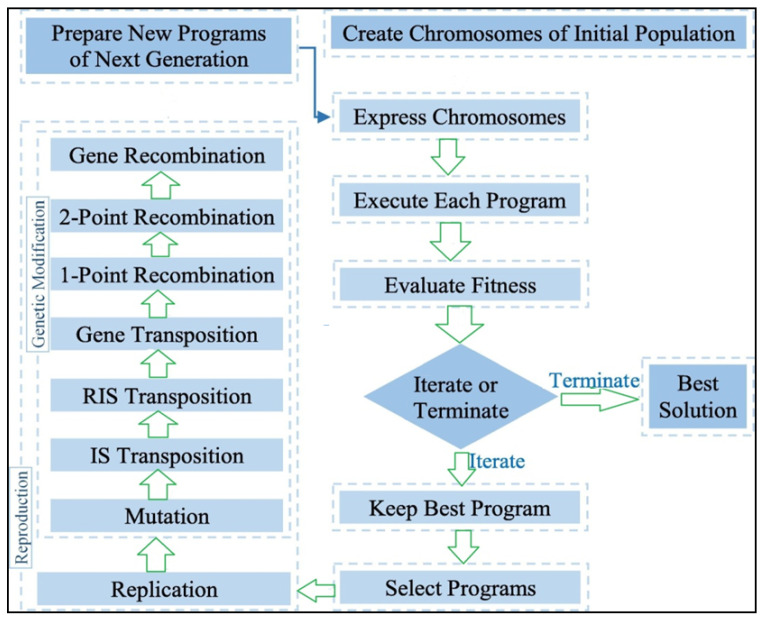
Scheme of gene expression programming.

GEP is a transformative algorithm that designs computer-based programs and models. These programs usually have a tree structure that is capable of modifying its size (size, shape and arrangement), like in the case of chromosomes. For this reason, GEP, being a genotype–phenotypic system, can be much more effective when compared to adaptive techniques. The programming language of GEP is known as Karva language and is the same as the LISP languages. The stages of GEP are shown in Figure 6. GEP has many advantages over other classical regression techniques, as, in other methods, some functions are initially defined and then analyzed. However, in GEP, no predefined function is taken into consideration.

Machine learning algorithms, such as ANNs and ensemble models, are successfully used to predict the concentration of ions in various conditions. It is possible to predict the chloride concentration in columns at different heights above the water level. In such cases, the error of each individual estimation is less than 20% [72]. Moreover, the service life of a concrete element can be modeled based on the chloride concentration at different depths in a sample. Linear regression was used for this purpose. The accuracy obtained by the linear correlation coefficient varied from 0.83 to 1.0 and was dependent on the zone in which the element was located [73]. In turn, ensemble models were used to predict the surface chloride concentration of marine concrete elements with good accuracy. This accuracy was proved by obtaining a relatively high value of the linear coefficient of correlation *R*^2^—equal to 0.83 [74]. Even though algorithms were previously used to predict the chloride concentration in concrete elements, there are no records of using gene expression programming for predicting chloride concentrations on the surface of marine concrete elements.

## 3. Results and Their Analyses

### 3.1. Statistical Analysis

**Figure 7:**
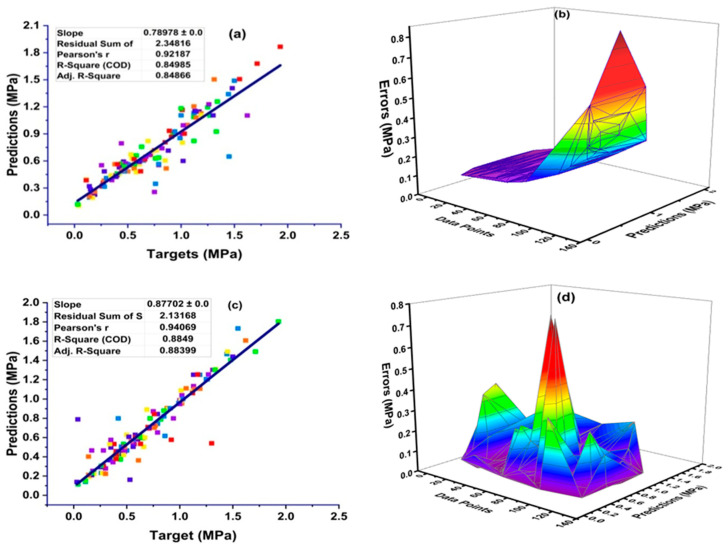

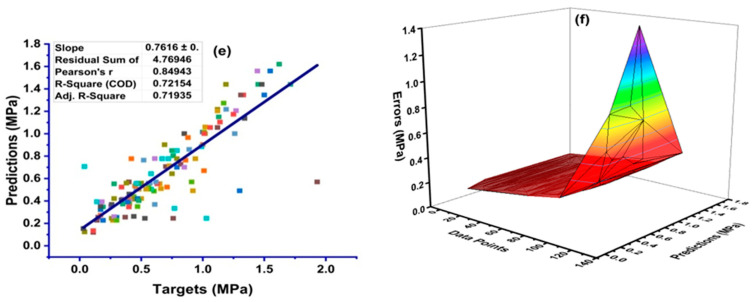
The results of the numerical analyses, which present the relationship between the predicted variable and the experimentally determined variable, as well as the distribution of errors for the ANN (**a**,**b**); GEP (**c**,**d**); and DT (**e**,**f**).

The results of the statistical analyses (presented as a relation between the measured value of *C_s_* and the value identified by the machine learning algorithms) and the error distribution charts are presented in Figure 7. ANN gives a strong relation in the form of R^2^ = 0.84, as can be seen in Figure 7a—with its error distribution shown in Figure 7b. The error distribution in Figure 7b illustrates that the average error of the training set is equal to 0.108 MPa. Moreover, the maximum and minimum error values of the training set were noted as 0.801 MPa and 0.0035 MPa, respectively. In addition, 69.1 percent of the data showed an error of less than 0.10 MPa, however, 64.3 percent of the data showed an error between 0.01 MPa and 0.10 MPa, as illustrated in Figure 7b.

The prediction of surface chloride concentration by employing the GEP algorithm yields a strong relationship between the targeted and output values of chloride concentrations, as shown in Figure 7c. It is also clear that this model gives a better response with less variance. GEP with an R^2^ value of 0.88 had a better accuracy when compared to the ANN (R^2^ equal to 0.84) and DT (R^2^ equal to 0.72), as depicted in Figure 7. In turn, Figure 7d indicates the error distribution of the linear regression model. It can be seen that 72.86 percent of the data showed an error between 0.01 MPa and 0.10 MPa, and that the average error of the training set was equal to 0.080 MPa. Moreover, the maximum and minimum errors were equal to 0.76 MPa and 0.004 MPa.

The influence of variables on the prediction of surface chloride concentrations using linear regression is illustrated in Figure 7e. The algorithm yields a decreased or poor correlation when predicting targets, which is indicated by the value of R^2^ being equal to 0.72, as shown in Figure 7e. In addition, Figure 7f presents the error distribution of the linear regression model and shows an average value of error equal to 0.12 MPa, with a maximum error value of 1.36 MPa. In turn, 82.1 percent of the model has an error between 0.005 MPa and 0.25 MPa.

### 3.2. K-Fold Cross Validation

The actual performance of the models was analyzed using the statistical cross-validation method. This method was used to evaluate the performance of the model. The K-fold validation test takes place in such a way that data are set randomly and split into k-groups. In this case, the data were divided into 10 groups, of which nine were used for training, and one was used for validation of the model. This was then repeated ten times in order to obtain the average value of these repetitions. When using the 10-fold cross-validation method, it is possible to obtain a relatively high performance of a model. Moreover, a statistical check was also applied to evaluate the model [72]. This statistical analysis is a check that shows the response of the model towards the prediction, as illustrated in the form of the equations listed below (Equations (2)–(5)).

(2) $$RMSE=\sqrt{\frac{\sum_{i=1}^{n}\;{\left(ex_{i}-mo_{i}\right)}^{2}}{n}}$$

(3) $$MAE=\frac{\sum_{i=1}^{n}|ex_{i}-mo_{i}|}{n}$$

(4) $$MSE=\frac{\sum_{i=1}^{n}{\left(mo_{i}-ex_{i}\right)}^{2}}{\sum_{i=1}^{n}{\left(\bar{ex}-ex_{i}\right)}^{2}}$$

(5) $$R=\frac{\sum_{i=1}^{n}\left(ex_{i}-{\bar{ex}}_{i}\right)\left(mo_{i}-{\bar{mo}}_{i}\right)}{\sqrt{\sum_{i=1}^{n}{\left(ex_{i}-{\bar{ex}}_{i}\right)}^{2}\sum_{i=1}^{n}{\left(mo_{i}-{\bar{mo}}_{i}\right)}^{2}}}$$

where,

$ex_{i}$—experimental value;
$mo_{i}$—predicted value;
${\bar{ex}}_{i}$—mean experimental value;
${\bar{mo}}_{i}$—mean predicted value obtained by the model;
*n*—number of samples.

**Figure 8:**
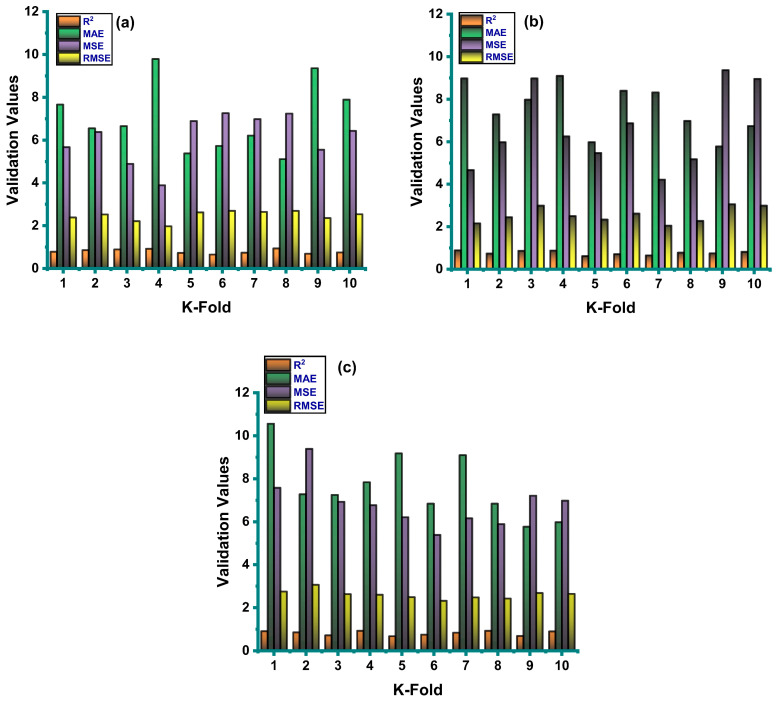
Statistical indicator for k-fold cross-validation. (**a**) GEP model. (**b**) ANN model. (**c**) DT model.

Correlation coefficient (R^2^), mean absolute error (MAE), mean square error (MSE), and root mean square error (RMSE) were all used to evaluate the result of cross-validation, as can be seen in Figure 8. A comparison of all three individual model techniques indicated fluctuation in their outputs. The GEP model showed fewer errors with a much better R^2^ value when compared to the ANN and DT. The average R^2^ value of GEP modeling was equal to 0.79, with maximum and minimum values being 0.94 and 0.65, as illustrated in Figure 8a. The average value of R^2^ for the ANN model was equal to 0.71, with the maximum and minimum values being 0.89 and 0.62. Similarly, the DT model gave an average R^2^ value of 0.82, with maximum and minimum values being 0.93 and 0.68, as illustrated in Figure 8b,c. The values of the errors of all the models were relatively low in the case of the validation process. For GEP, they were: MAE = 7.03 MPa, MSE = 6.12 MPa, and RMSE = 2.46 MPa (Figure 8a). In the case of the ANN, they were: MAE = 7.56 MPa, MSE = 6.60 MPa, and RMSE = 2.54 MPa (Figure 8b); and in the case of the decision tree, they were: MAE = 7.66 MPa, MSE = 6.85 MPa, and RMSE = 2.61 MPa (Figure 8c). Moreover, the K-fold cross validation of all the applied models and statistical checks are listed in Table 4 and Table 5, respectively.

## 4. Discussion

**Figure 9:**
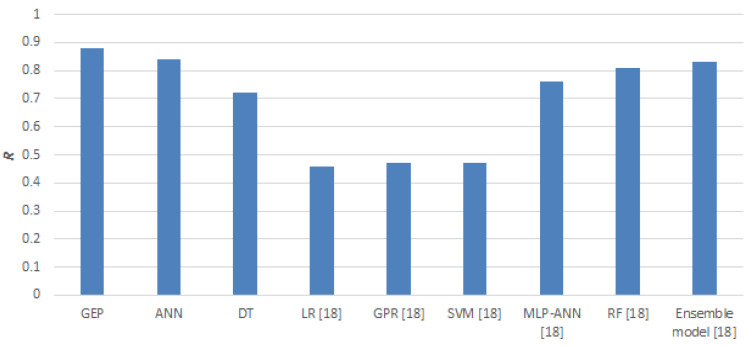
Comparison of the accuracy of different machine learning algorithms.

This research describes the predictive performance of chloride concentrations on the surface of marine structures using individual supervised machine learning algorithms. The three machine learning algorithms used during the investigation were: artificial neural network, decision tree, and gene express programming. GEP, with an R^2^ value of 0.88, was the most accurate when compared to the ANN (R^2^ equal to 0.84) and DT (R^2^ equal to 0.72). This algorithm was also compared with those used in [18], and the results of the comparison accuracy are presented in Figure 9.

It can be seen from Figure 9 that the proposed GEP algorithm accurately describes chloride surface concentrations when compared to other algorithms. This is confirmed by the very high value of linear correlation coefficient R^2^, which is on a comparable level to other algorithms used in the literature.

## 5. Conclusions

This research describes the predictive performance of chloride concentrations on the surface of marine structures using individual supervised machine learning algorithms. The three algorithms—the artificial neural network, the decision tree, and gene expression programming—were used for the investigations. The most accurate among these three was GEP, which was proved by the fact that it obtained the highest value of the linear correlation coefficient and the lowest values of the parameters describing the errors of prediction. The following conclusions can be drawn:

● The GEP algorithm is very effective for predicting chloride surface concentrations and can be successfully used for this purpose. This was also proved by comparing it with other algorithms used in the literature.
● The presented method does not depend on the zone in which it is used (except the atmospheric zone where the transport of chloride ions is more difficult to describe).
● The high performance of the GEP algorithm was also proved using k-fold validation.

The chloride surface concentration model, which uses gene expression programming, was proposed in this work. It can be successfully used without the need of investing significant time and money, as is the case with long term experiments. However, there is still room for improvement:

● The dataset can be expanded with laboratory tests, field tests, or numerical analyses using different upsizing methods (e.g., Monte Carlo).
● There is still the possibility of expanding the dataset with the results of surface chloride concentrations obtained for elements located in the atmospheric zone.
● Due to the fact that there is no model in the literature that is 100% accurate, there is still the possibility of using a different, more accurate, algorithm.

## Figures and Tables

**Table 1:** Prediction properties with different approaches.

Lp.	Algorithm Name	Notation	Dataset	Prediction Properties	Year	Waste Material Used	References
1.	Individual with ensemble modeling	ANN, bagging and boosting	1030	Compressive strength	2021	FA	[28]
2.	Data envelopment analysis	DEA	114	Compressive strength Slump test L-box test V-funnel test	2021	FA	[29]
3.	Multivariate	MV	21	Compressive strength	2020	Crumb rubber with SF	[30]
4.	Support vector machine	SVM	-	Compressive strength	2020	FA	[31]
5.	Support vector machine	SVM	115	Slump test L-box test V-funnel test Compressive strength	2020	FA	[32]
6.	Adaptive neuro fuzzy inference system	ANFIS with ANN	7	Compressive strength	2020	POFA	[33]
7.	Gene expression programming	GEP	277	Axial capacity	2020	-	[34]
8.	Gene expression programming	GEP	357	Compressive strength	2020	-	[35]
9.	Random forest and gene expression programming	RF and GEP	357	Compressive strength	2020	-	[36]
10.	Artificial neural network	ANN	205	Compressive strength	2019	FA GGBFS SF RHA	[37]
11.	Intelligent rule-based enhanced multiclass support vector machine and fuzzy rules	IREMSVM-FR with RSM	114	Compressive strength	2019	FA	[38]
12.	Random forest	RF	131	Compressive strength	2019	FA GGBFS FA	[39]
13.	Multivariate adaptive regression spline	M5 MARS	114	Compressive strength Slump test L-box test V-funnel test	2018	FA	[40]
14.	Random kitchen sink algorithm	RKSA	40	V-funnel test J-ring test Slump test Compressive strength	2018	FA	[41]
15.	Adaptive neuro fuzzy inference system	ANFIS	55	Compressive strength	2018	-	[42]
16.	Artificial neural network	ANN	114	Compressive strength	2017	FA	[43]
17.	Artificial neural network	ANN	69	Compressive strength	2017	FA	[44]
18.	Artificial neural network	ANN	169	Compressive strength	2016	FA GGBFS FA RHA	[45]
19.	Artificial neural network	ANN	80	Compressive strength	2011	FA	[46]
20.	Artificial neural network	ANN	300	Compressive strength	2009	FA	[47]

**Table 2:** Range of input and output variables.

Parameters	Abbreviation	Units	Minimum	Maximum
Cement	C	kg/m^3^	110	519
Fly ash	FA	kg/m^3^	0	239
Ground furnace slag	GFS	kg/m^3^	0	292.5
Silica fume	SF	kg/m^3^	0	50
Super plasticizer	SP	kg/m^3^	0	10.2
Water	W	kg/m^3^	38.5	311
Fine aggregate	FA	kg/m^3^	552	1232
Coarse aggregate	CA	kg/m^3^	410	1305
Water/binder	W/C	-	0.3	0.75
Exposure time	T	years	0.08	48.65
Annual mean temperature	AMT	°C	7	50
Chloride concentration in seawater	CCS	kg/m^3^	13	27.37
Surface chloride concentration	SCC	kg/m^3^	0.023	1.945

**Table 3:** Descriptive analysis of parameters.

Parameters Descriptionr	C	FA	GFS	SF	SP	W	FA	CA	W/C	T	AMT	CCS
Mean	370.70	33.97	11.41	5.41	1.47	187.54	765.77	993.81	0.46	4.24	17.78	18.99
Standard Error	2.98	2.36	1.79	0.51	0.08	1.74	4.60	5.35	0.00	0.25	0.37	0.11
Median	375.00	0.00	0.00	0.00	1.00	180.00	800.00	1020.00	0.45	2.67	16.80	19.00
Mode	340.00	0.00	0.00	0.00	1.00	180.00	800.00	1020.00	0.45	5.00	7.00	19.00
Standard Deviation	75.61	59.88	45.45	12.85	1.99	44.08	116.46	135.43	0.08	6.28	9.38	2.79
Sample Variance	5716.21	3585.21	2066.15	165.02	3.95	1943.32	13,563.76	18,342.52	0.01	39.46	88.06	7.81
Kurtosis	0.60	3.46	15.48	4.11	4.67	2.80	4.74	7.56	1.04	24.71	−0.85	0.72
Skewness	−0.65	1.99	4.03	2.29	2.09	0.82	1.46	−1.85	0.96	4.45	0.42	0.52
Range	409.00	239.00	292.50	50.00	10.20	272.50	680.00	895.00	0.45	48.57	43.00	14.37
Minimum	110.00	0.00	0.00	0.00	0.00	38.50	552.00	410.00	0.30	0.08	7.00	13.00
Maximum	519.00	239.00	292.50	50.00	10.20	311.00	1232.00	1305.00	0.75	48.65	50.00	27.37
Sum	237,992.50	21,809.50	7324.00	3475.00	942.56	120,398.80	491,625.00	638,027.00	292.43	2720.83	11,417.40	12,190.73
Count	642.00	642.00	642.00	642.00	642.00	642.00	642.00	642.00	642.00	642.00	642.00	642.00

**Table 4:** Results of k-fold cross validation.

K-Fold	GEP				ANN				DT			
	R^2^	MAE	MSE	RMSE	R^2^	MAE	MSE	RMSE	R^2^	MAE	MSE	RMSE
1	0.78	7.66	5.67	2.38	0.89	8.98	4.67	2.16	0.91	10.56	7.58	2.75
2	0.86	6.55	6.38	2.53	0.74	7.29	5.98	2.45	0.86	7.28	9.39	3.06
3	0.89	6.65	4.89	2.21	0.87	7.98	8.98	3.00	0.72	7.25	6.93	2.63
4	0.92	9.79	3.89	1.97	0.88	9.10	6.25	2.50	0.93	7.84	6.77	2.60
5	0.73	5.38	6.89	2.62	0.62	5.98	5.47	2.34	0.68	9.18	6.21	2.49
6	0.65	5.73	7.26	2.69	0.71	8.40	6.87	2.62	0.75	6.84	5.39	2.32
7	0.74	6.21	6.98	2.64	0.65	8.32	4.22	2.05	0.84	9.10	6.16	2.48
8	0.94	5.11	7.24	2.69	0.78	6.98	5.18	2.28	0.93	6.84	5.89	2.43
9	0.69	9.36	5.55	2.36	0.75	5.78	9.37	3.06	0.69	5.77	7.21	2.69
10	0.75	7.89	6.43	2.54	0.82	6.74	8.96	2.99	0.90	5.98	6.98	2.64

**Table 5:** Statistical checks.

Machine Learning Methods	MAE (MPa)	MSE (MPa)	RMSE (MPa)
Gene Expression Program	4.36	28.51	5.33
Artificial Neural Network	4.48	31.86	5.64
Decision Tree	4.55	36.73	6.06

## Data Availability

The data presented in this article are available within the article.

## Abbreviations

**Notations**	
ML	Machine learning
HPC	High performance concrete
GGBS	Ground granulated blast furnace slag
GEP	Genetic engineering programming
ANN	Artificial neural network
Dt	Decision tree
DL	Deep learning
DM	Deep machine
RF	Random forest
GB	Gradient boosting
FA	Fly ash
SF	Silica fume
POFA	Palm oil fuel ash
SCC	Self-compacting concrete
RHA	Rice husk ash
RKSA	Random kitchen sink algorithm
SVM	Support vector machine
ANFIS	Adaptive neuro fuzzy inference system
IREMSVM-FR	Intelligent rule-based enhanced multiclass support vector machine
RMS	Response surface method
